# How sensitive are estimates of carbon fixation in agricultural models to input data?

**DOI:** 10.1186/1750-0680-7-3

**Published:** 2012-02-01

**Authors:** Markus Tum, Franziska Strauss, Ian McCallum, Kurt Günther, Erwin Schmid

**Affiliations:** 1 Deutsches Zentrum für Luft- und Raumfahrt (DLR), Deutsches Fernerkundungsdatenzentrum (DFD), Oberpfaffenhofen, D-82234 Wessling, Germany; 2 University of Natural Resources and Life Sciences Vienna, Feistmantelstrasse 4, A-1180 Vienna, Austria; 3 International Institute for Applied Systems Analysis, Schlossplatz 1, A-2361 Laxenburg, Austria

**Keywords:** agricultural models, net primary productivity, EPIC, BETHY/DLR, land cover, weather

## Abstract

**Background:**

Process based vegetation models are central to understand the hydrological and carbon cycle. To achieve useful results at regional to global scales, such models require various input data from a wide range of earth observations. Since the geographical extent of these datasets varies from local to global scale, data quality and validity is of major interest when they are chosen for use. It is important to assess the effect of different input datasets in terms of quality to model outputs. In this article, we reflect on both: the uncertainty in input data and the reliability of model results. For our case study analysis we selected the Marchfeld region in Austria. We used independent meteorological datasets from the Central Institute for Meteorology and Geodynamics and the European Centre for Medium-Range Weather Forecasts (ECMWF). Land cover / land use information was taken from the GLC2000 and the CORINE 2000 products.

**Results:**

For our case study analysis we selected two different process based models: the Environmental Policy Integrated Climate (EPIC) and the Biosphere Energy Transfer Hydrology (BETHY/DLR) model. Both process models show a congruent pattern to changes in input data. The annual variability of NPP reaches 36% for BETHY/DLR and 39% for EPIC when changing major input datasets. However, EPIC is less sensitive to meteorological input data than BETHY/DLR. The ECMWF maximum temperatures show a systematic pattern. Temperatures above 20°C are overestimated, whereas temperatures below 20°C are underestimated, resulting in an overall underestimation of NPP in both models. Besides, BETHY/DLR is sensitive to the choice and accuracy of the land cover product.

**Discussion:**

This study shows that the impact of input data uncertainty on modelling results need to be assessed: whenever the models are applied under new conditions, local data should be used for both input and result comparison.

## Background

Modelling the net carbon uptake by vegetation (Net Primary Productivity, NPP) and estimating the yields of agricultural plants have become important tools to study the mechanisms of carbon exchange between the atmosphere and vegetation, as well as issues of food security. Different approaches are currently tracked which can be grouped to their approaches how photosynthesis is modelled.

Models describing the chemical, physical and plant physiological processes of plant development and the interaction of plants with the atmosphere can be applied to simulate the rate of carbon dioxide uptake of the plant through photosynthesis (called Gross Primary Productivity, GPP). These models follow the concept of [1] and [2] to simulate the process of photosynthesis. Moreover, carbon uptake of well-watered and fertilized annual plants is linearly related to the amount of absorbed Photosynthetically Active Radiation (PAR), which can be derived from satellite data (i.e. the fraction of PAR which is absorbed by the canopy; cp. [3] or calculated by the accumulation of dry matter.

NPP is defined as the difference between GPP and autotrophic respiration. Therefore, it is important to estimate the autotrophic respiration of plants following the determination of GPP. Autotrophic respiration is defined as the oxidation of organic compounds found in roots, stems and leaves, to CO_2 _or water. In the literature, different approaches to estimate autotrophic respiration are discussed, taking into account the actual biomass or GPP (e.g. [4-6]). When the Light Use Efficiency (LUE) approach is integrated in a coupled soil - plant - atmosphere model as in the EPIC (Environment Policy Integrated Climate) model, daily estimates of evapotranspiration and carbon assimilation fluxes can be obtained [7].

In contrast to these models, more sophisticated approaches are in use and under development. These models track photosynthesis on the molecule level. They take into account the interaction between plants, atmosphere and soil by simulating the uptake and release of carbon by plants and soil in a physically consistent way including conservation of energy and momentum.

In the literature one can find descriptions of established vegetation models for use on different scales [8-11]. Each of these models is driven by meteorological input data and parameterized for global use with special focus on the long-term competition between the plant functional types when natural disturbance and succession driven by light competition occur. Models with a spatial resolution of kilometres and a time horizon of some years as e.g. the soil-vegetation-atmosphere-transfer (SVAT) model BETHY/DLR (Biosphere Energy Transfer Hydrology Model) [12] which can be used for regional assessments of NPP or biomass development.

During the last decades, the use of both modelling approaches was often met with resistance, mainly because of the need of calibration, validation and determination of the level of uncertainty (e.g.: [13-15]). Furthermore for many users, i.e. policy makers, it is difficult to judge whether the model outputs are within acceptable levels of uncertainty or not, mainly due to their limited background in model development [16]. However, in this context it is of importance to the policy maker to understand the validity of the model results and their associated uncertainties.

Since empirical research traditionally advances in its data accuracy and validity - in contrast - process-based models do not always provide comparable outputs, it is difficult to judge on the quality of modelled data, especially with the traditional criteria for assessing scientific outcomes [17]. However, regardless of the data's source, there will always be some uncertainty associated with it.

To address these issues, we have assessed the variability of the soil-vegetation-atmosphere-transfer model BETHY/DLR [12] and the bio-physical process model EPIC [7] on three different meteorological input datasets and two land cover maps. Since the two models were designed for different specific purposes, we do not intend to discuss advantages or disadvantages but place special attention on the investigation of model sensitivity to the spatial resolution of the input datasets. The Austrian Marchfeld region has been chosen as case study analysis because many datasets (Table 1) are readily available. The period of investigation is 2000 to 2003. It is important to note that this study is not a classical sensitivity analysis for assessing systematically the responses of models to changes in input data and model parameters (e.g. [18-21]), but a model variability analysis.

**Table 1:** Meteorological, land cover, and other data.

Datatype	Period used	Resolution of space and time	Parameters used	Characteristics	References
Meteorology	2000-2003	weather stations; daily	Precipitation; Minimum temperature; Maximum temperature; Wind Speed; Radiation; Relative Humidity	Measured time series	Central Institute for Meteorology and Geodynamics (ZAMG)
Meteorology	2000-2003	1 km^2 ^grid; daily	Precipitation; Minimum temperature; Maximum temperature; Wind Speed; Radiation; Relative Humidity	Reallocated time series (from now on 'ZAMG reallocated')	[32]
Meteorology	2000-2003	0.25°; up to 4 times a day	Precipitation; Minimum temperature; Maximum temperature; Wind Speed; Cloud cover; Soil Water Content	Time series of model re-analysis (ERA-40)	European Centre for Medium-Range Weather Forecasts (ECMWF)
Vegetation Indices	2000-2003	1 km^2 ^grid; 36 time steps per year	Leaf Area Index (LAI)	Global coverage	Pôle d'Observation des Surfaces continentales par TELédétection (POSTEL)
Landcover	2000	1 km^2 ^grid, year 2000	Land cover / land use information; 22 classes	Global coverage (GLC2000)	[35,36]
Landcover	2000	1 ha grid, year 2000	Land cover / land use information; 44 classes	European coverage (CORINE 2000)	[37]
Census	1999 'Agrar-struktur-erhebung'	Marchfeld sub-regions, year 1999	Agricultural land use information; Main soil type distribution	Land use data of farms aggregated to municipalities	[30,42]

## Methods

### Biophysical process models

EPIC is a comprehensive model under continuous development since 1981, capable of simulating many agricultural processes that occur as a result of climate forcing, landscape characteristics, soil conditions and crop management schemes [7,22,23]. Biophysical processes simulated with EPIC include among others plant and crop growth, hydrology, wind and water erosion, and nutrient cycling. These processes are simulated with daily time steps or smaller. EPIC contains algorithms that allow for a complete description of the hydrological balance at the small watershed scale (up to 100 ha) including snowmelt, surface runoff, infiltration, soil water content, percolation, lateral flow, water table dynamics, and evapotranspiration. Daily weather can be endogenously generated for precipitation, temperature, solar radiation, wind speed, and relative humidity or it can be input exogenously.

EPIC uses the concept of radiation-use efficiency by which a fraction of daily photosynthetically active radiation is intercepted by the plant canopy and converted into plant biomass. The leaf area index is simulated as a function of heat units, crop stress and development stages. Daily gains in plant biomass are affected by vapor pressure deficits and atmospheric CO_2 _concentration [24]. Crop yield is simulated using the harvest index which is affected by the heat unit factor and includes the amount of the crop removed from the field as well as the above-ground biomass. Stress indices for water, temperature, nitrogen, phosphorus and aeration are calculated daily using the value of the most severe of these stresses to reduce potential plant growth and crop yield. Similarly, stress factors for soil strength, temperature, and aluminum toxicity are used to adjust potential root growth [25].

The soil water balance depending on the potential water use, the root zone depth and the water use distribution parameter is applied in a general water use function where any water deficit can be overcome if a layer that is encountered has adequate water storage. The potential water use is reduced when the soil water storage is less than 25% of plant-available soil water by using dependencies on the soil water contents at field capacity and wilting point [7].

BETHY/DLR belongs to the family of SVAT models, which track the transformation of atmospheric carbon dioxide into energy storing sugars, a process known as photosynthesis. BETHY/DLR is based on the Jena Scheme of Atmosphere Biosphere Coupling in Hamburg (JSBACH) by [4] and was modified by [12]. The JSBACH model was originally considered for global usage and computes the biosphere-atmosphere exchange within the Global Circulation Model ECHAM5 (European Centre Hamburg). BETHY/DLR as well as JSBACH use the combined approach to integrate photosynthesis [26,27], which means that the enzyme kinetics are parameterized on the leaf level. In this context, C3 and C4 plants are distinguished because of significant differences in the way of their carbon-fixation: C4 plants (e.g. corn and sugar cane) are able to fix more atmospheric carbon dioxide at high temperatures than C3 plants (e.g. wheat and barley). Thus, the photosynthesis of C3 plants is saturated at higher temperatures. In a second step, the rate of photosynthesis is extrapolated from leaf to canopy level by taking into account both, the canopy structure as well as the interaction of the plant between soil, atmosphere and vegetation. The two-flux scheme of [28] which includes three canopy layers, is used to approximate the radiation absorption in the canopy. Evapotranspiration, stomatal conductance and the soil water balance is included in the model formulation. To compute NPP on an annual basis snow is included in the water budget. Water stress is considered by calculating the demand for evapotranspiration using the approach of [2] limited by the criteria of [29]. Here it is assumed, that evapotranspiration can not be higher than a certain soil water supply via roots. Autotrophic respiration is evaluated as the sum of maintenance and growth respiration. The plant specific dark respiration determines the maintenance respiration, while growth respiration is assumed to be proportional to the difference between GPP and maintenance respiration. The main outputs of BETHY/DLR are given by time series of GPP, NPP, evapotranspiration, and of soil water content in daily steps with the spatial resolution of the respective land cover classification. A more detailed model description can be found in [12].

The general characteristics as e.g. main outputs and the general formulation to compute NPP of the two models BETHY/DLR and EPIC are presented in table 2.

**Table 2:** General characteristics of the biophysical process models EPIC and BETHY/DLR.

	BETHY/DLR	EPIC
Abbreviation	Biosphere Energy Transfer Hydrology Model	Environmental Policy Integrated Climate
References	[4,12]	[7,22,23]
Model type	SVAT model	crop model
Time step	Daily	Up to < 1 day
Main simulation processes	GPP, NPP, NEP, evapotranspiration, soil water content	plant and crop growth, heat and water balance, wind and water erosion, nutrient cycling
General formulation to compute NPP	NPP = GPP - autotrophic respiration	NPP = (yield+straw+roots)- (water content+non carbon fraction)

### Framework of Case Study Analysis

**Figure 1:**
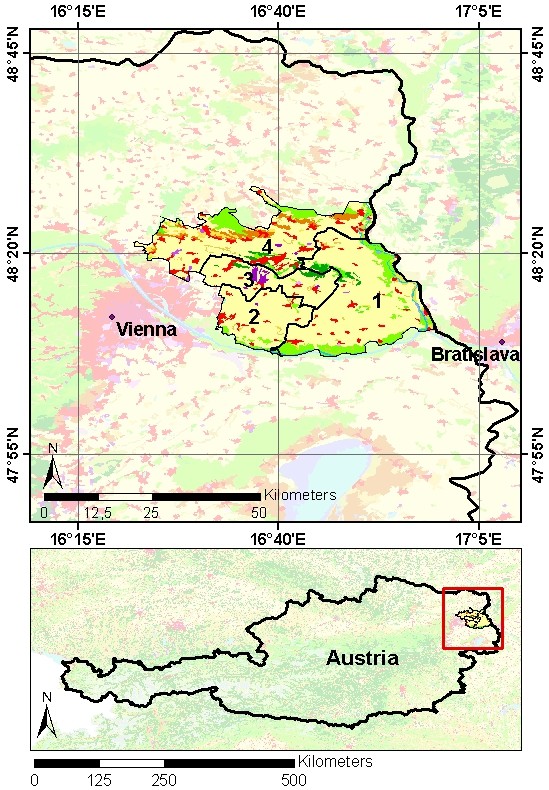
**Map of the study area**. The case study area Marchfeld with the four sub-regions (upper figure), with underlying CORINE land cover dataset 2000. Green pixels represent forest, red and violet pixels urban areas, brown pixels shrub land, and yellow pixels agricultural areas. The lower figure highlights the location of the Marchfeld region. The red square represents the map extract of the upper figure.

The Austrian Marchfeld region serves as case study area to assess the variability of the two biophysical process models on alternative input datasets. The EPIC model has already been applied and validated here [30], and the data necessary for our study is readily available (see table 1). The Marchfeld region is located in Lower Austria, part of the Vienna Basin, and forms with around 100,000 ha one of the largest plains in Austria. Around 75% of the area is used for agricultural production. The natural boundaries are to the East the river March (the Austrian border to Slovakia), to the North the hills of the Weinviertel, to the West the mountain range of Bisamberg and the city of Vienna, and to the South the river Danube. For locating the region a map is presented in Figure 1.

Since land use practices are not homogenously distributed in this area, five sub-regions have been identified using the cluster analysis methods [31]. Each sub-region has an area of in between 85 km^2 ^and 250 km^2 ^. The urban land cover as well as forest and shrub lands have not been taken into account in the variability analysis. Five typical soils have been selected with respect to majority criteria for the agricultural land cover (four different Chernozems and one black earth; [30]).

The biophysical process models have been applied with different meteorological inputs (table 1) from the period 2000 to 2003. We have used meteorological observations from weather stations of the Central Institute for Meteorology and Geodynamics (ZAMG) in the Marchfeld region, reallocated meteorological data from weather stations across Austria of ZAMG [32], and meteorological data from the European Centre for Medium-Range Weather Forecasts (ECMWF).

The meteorological observations (ZAMG) are from the weather station in Gross Enzersdorf, and provide daily values of six weather parameters including minimum and maximum temperatures, relative humidity, wind speed precipitation and solar radiation.

[32] developed a reallocated meteorological dataset comprising climate data for Austria and the period from 1975 to 2007 with temporal and spatial resolutions of one day and 1 km^2 ^. In addition climate change scenarios have been developed for the period 2008 to 2040. They processed daily data from 34 weather stations of ZAMG to 60 spatial climate clusters with homogeneous climates relating to mean annual precipitation sums and mean annual temperatures from the period 1961-1990. Based on these precipitation and temperature classes four climate clusters describe the climate in the Marchfeld region (cluster 1: mean annual precipitation sums smaller than 500 mm and mean annual temperatures between 8.5°C and 9.5°C; cluster 2: mean annual precipitation sums smaller than 500 mm and mean annual temperatures between 9.5°C and 10.5°C; cluster 3: mean annual precipitation sums between 500 mm and 600 mm and mean annual temperatures between 8.5°C and 9.5°C; cluster 4: mean annual precipitation sums between 500 mm and 600 mm and mean annual temperatures between 9.5°C and 10.5°C). For each homogenous climate cluster, [32] performed regression model analyses primarily to compute a set of daily climate data for the time period 2008 to 2040. This method has also been applied for the time period 1975 to 2007 to provide a consistent dataset. The integral parts of the regression model are i) the consideration (extrapolation in the period 2008 to 2040, respectively) of the observed linear temperature trend from 1975 to 2007 derived from a homogenized dataset, and ii) the repeated bootstrapping of temperature residuals and of observations for solar radiation, precipitation, relative humidity, and wind speed to ensure consistent spatial and temporal correlations. We have also used these reallocated data for the period 2000 to 2003 in our variability analysis.

The third dataset is derived from ECMWF data and has a temporal resolution of up to four times a day and a spatial resolution of 0.25° × 0.25°. It includes model analysis data of 2 m air temperature, cloud cover, soil water content of the four upper layers and wind speed at 10 m above ground. From this dataset the daily mean, as well as minimum and maximum temperatures and the daily mean of cloud cover in all three strata (high, medium, low) are used. The daily temperature values are scaled with the difference between ECMWF reference height and the global ETOP05 (Earth Topography and Ocean Bathymetry Database) 5-minute gridded elevation data by using the temperature gradient of the U.S. Standard Atmosphere (-0.65 K per 100 m) in order to downscale the ECMWF temperature data to km^2 ^resolution. Precipitation values are derived twice a day from the ECMWF re-analysis project (ERA-40). PAR is not used directly from the corresponding ECMWF product data as it is only available as forecast data and therefore rather uncertain. Thus, daily PAR is determined from global radiation which is computed following the approach of [33] taking into account the geographical coordinates of the day, and using a transmission, which depends on the degree of cloudiness. The degree of cloudiness is calculated as a weighted sum of each cloud strata for each day, and the global radiation is calculated for each location in the time step of one hour. The advantage of this approach is the use of analysis data of cloud coverage to compute PAR data which leads to more exact results than directly using the PAR forecast data [12].

Hence the BETHY/DLR model needs an initial soil water content, the ECMWF soil water dataset is used only for the transient phase of the model. Afterwards the model simulates the soil water content independently, according to the hydrological boundary conditions. Investigations of [12] have shown that in most cases sufficient hydrological boundary conditions are available after a transient phase of about one year.

In addition to the meteorological data, the BETHY/DLR model is driven by two sets of remote sensing data. Detailed and homogenous land cover / land use information are used to get information about the vegetation types the model is run for. Vegetation is represented by time series of the Leaf Area Index (LAI). Time series of LAI were used from the "Carbon cycle and Change in Land Observational Products from an Ensemble of Satellites" (CYCLOPES) 10 day composite datasets of POSTEL (Pole d'Observation des Surfaces continentales par TELedetection), which have a spatial resolution of 1 km × 1 km. For each of the grid cells, time series analysis has been applied in order to eliminate data gaps and outliers. In the framework of this study the harmonic analysis has been used. The method of the harmonic analysis is based on the method of superposition such as the Fourier transformation. This method ([34]) is used to process LAI time series at the German Remote Sensing Data Center.

The CYCLOPES dataset additionally contains information of land cover and land use and is available as GLC2000 (valid for the year 2000). The Land Cover Classification System of the Food and Agriculture Organization of the United Nations has been used to derive land cover classes of GLC2000 resulting in 22 different land cover classes [35,36].

A translation of the GLC2000 vegetation classes had to be performed in order to use the GLC2000 land use / land cover classification to model NPP with BETHY/DLR. The actual model setup of BETHY/DLR includes 33 inherent vegetation classes which can be regarded as vegetation types. Each vegetation type is linked with biochemical parameters as i.e. the maximum electron transport rate and the maximum carboxylation rate, and other vegetation specific parameters as maximum height and rooting depth. These parameters describe the mechanism of photosynthesis of vegetation. In this study only the GLC2000 class 16 "Cultivated and managed areas" has been used and translated to the BETHY/DLR vegetation type "arable land" as no further detailed information about the land use (e.g. crop rotation) is available from the GLC2000.

In addition to the GLC2000 dataset the Coordinated Information on the European Environment (CORINE) 2000 land cover / land use classification has been used, to validate the GLC2000 dataset. The CORINE 2000 data was derived from LANDSAT satellite images and is also available for the year 2000 [37]. The CORINE 2000 is available as raster datasets in spatial resolutions of 100 m × 100 m, 250 m × 250 m and 1 km × 1 km for 32 European countries, including Austria. For this study the dataset with resolution 100 m × 100 m has been used. The CORINE 2000 provides information about 44 vegetation classes which had also to be translated to BETHY/DLR vegetation types. We assumed that only the CORINE 2000 class "Non-irrigated arable land" contains the needed information about agricultural land, since all other classes which are available for the Marchfeld region report different land use (e.g. forests and urban areas). The CORINE 2000 class "Non-irrigated arable land" is then translated to the BETHY/DLR class "arable land".

### From Crop Yield to NPP

The crop yields of EPIC for the thirteen crops in the Marchfeld region have been converted to NPP values (table 2) for comparison with the BETHY/DLR outputs, which are given as time series of NPP. For this purpose, conversion factors of the relation between yield and straw as well as the above- and below- ground biomass are used. Empirical conversion factors about the relations between crop yield and straw yield can be found in e.g. [38,39]. In a first step, the above-ground biomass is computed for each crop using these empirical conversion factors. In a second step the below-ground biomass is computed with the use of conversion factors about the ratio of above- to below- ground biomass which are described in [40]. These conversion factors which originally have been derived for crops in Canada are assumed to be valid for the area of interest as well, as it already was proposed by [41]. After calculating the biomass of the whole plant, the remaining water content and the non carbon content have to be subtracted, following crop specific values, which are also reported in e.g. [38]. A detailed description of the approach and the used factors can be found in [41].

In order to compare the now available NPP per crop and sub-regions of EPIC with the BETHY/DLR results, statistical data about the land use of each of the four sub-regions is used to aggregate the NPP of EPIC. These statistical data provided by [30] and [42] give detailed information about the distribution of agricultural area over the thirteen main crops as well as the distribution of the five main soils being representative for the Marchfeld region. The results of BETHY/DLR have been aggregated to annual sums per sub-region with a Geographic Information System (GIS) tool, taking into account the equi-rectangular projection (latitude - longitude, WGS84 (World Geodetic System 1984)) of the data.

## Results and discussion

The variability analysis consists of seven model setups to compare model response to different input datasets. Three model simulations with the EPIC model have been performed and four with the BETHY/DLR model. The model setups are presented in table 3.

**Table 3:** Model setups for the variability analysis

Model	Meteorological input	Land cover classification	Short Name
BETHY/DLR	ZAMG	CORINE 2000	BETHY(1)
	ZAMG reallocated	CORINE 2000	BETHY(2)
	ECMWF	CORINE 2000	BETHY(3)
	ECMWF	GLC2000	BETHY(4)
EPIC	ZAMG	-	EPIC(1)
	ZAMG reallocated	-	EPIC(2)
	ECMWF	-	EPIC(3)

The EPIC model requires homogeneity with respect to data input (i.e. soil, topography, weather, crop management) such that the model has been applied for all combinations of climate, soil, and crop management, separately. Thus, the variability analysis has been conducted mainly for the meteorological datasets. In total 60 different model runs have been performed with EPIC for each crop. In contrast, the BETHY/DLR model is driven with the two different land cover classifications as well as the three different meteorological input data sets. For the Marchfeld region the FAO soil map of the world, which is used as input data for BETHY/DLR, reports one major soil type (Haplic Chermozem) which occupies 89% of the area and four additional soil types for the rest of the area. The EPIC model setup EPIC(1) is interpreted as reference, as it represents the already validated model setup [30].

**Figure 2:**
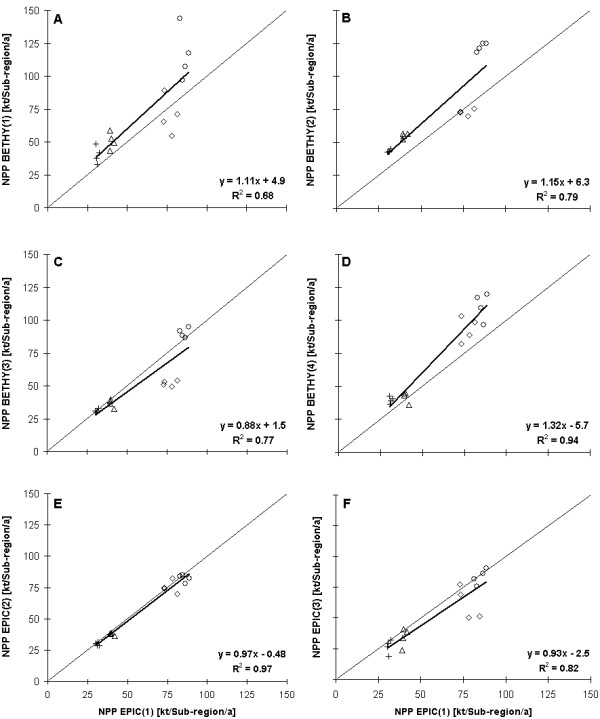
**Comparison of the model results**. Comparison of the model results (NPP) of BETHY/DLR and EPIC for the four Marchfeld sub-regions and the period 2000 to 2003. The nomenclature follows the scheme of table 3. Circles represent sub-region 1, triangles sub-region 2, crosses sub-region 3 and diamonds sub-region 4.

In Figure 2, all model results fare compared to the EPIC(1) results (table 3). The values of NPP are given in kilotonnes carbon per sub-region and year.

Depending on the model setup, the NPP results of BETHY/DLR show a variability of overestimations of up to 32% and underestimations of up to 12%, linked with coefficients of determination between 0.94 and 0.63, respectively. The highest overestimation of NPP (32%) is modelled when using the GLC2000 and meteorological input data from ECMWF (Figure 2D). Figure 2D represents the results of both models with the typical setup which was used in previous investigations (default setup). This overestimation is combined with a high coefficient of determination of about 0.94. When changing the land cover classification from GLC2000 to CORINE 2000 (while the meteorological input remains unchanged) an underestimation of about 12% has been found (Figure 2C). From Figure 2C it is evident that only 4 BETHY/DLR results determine the underestimation and thus the coefficient of determination of about 0.77. These four data points are all representative for sub-region 4, whereas the rest is close to the 45° line. Using measured meteorological data from ZAMG results in an overestimation of NPP of about 11% (Figure 2A), which is combined with the highest variability within the sub-regions and years for all four model setups of BETHY/DLR. Nevertheless a high coefficient of determination of about 0.68 is achieved. When using the reallocated ZAMG data of [32] for BETHY/DLR combined with CORINE as land cover an overestimation of the modelled NPP of about 15% (Figure 2B) has been found. A strong correlation of the simulation years is observed, which indicates homogeneity in the meteorological data.

The comparison between EPIC results with different weather input reveals that the ECMWF data affects the EPIC model to underestimate NPP by 8% (Figure 2F). The use of the reallocated meteorological dataset (Figure 2E) results in a little underestimation, linked with the highest coefficient of determination (0.97). Figure 2E demonstrates that EPIC is not very sensitive to measured or homogenized meteorological input data just in contrast to BETHY/DLR which can be seen in Figure 2A and 2B. Measured meteorological data during the four years result in a high variability of the annual NPP of sub-regions 1 and 4 while the reallocated meteorological data cluster the annual NPP of all sub-regions resulting in low variability for all sub-regions.

Figure 2D and figure2F show that the EPIC model as well as the BETHY/DLR model react in a similar way when alternating between ECMWF and ZAMG data. The BETHY/DLR model simulates 23% more NPP when using the ZAMG data, and the EPIC model simulates around 7% more NPP when using the ZAMG data.

**Figure 3:**
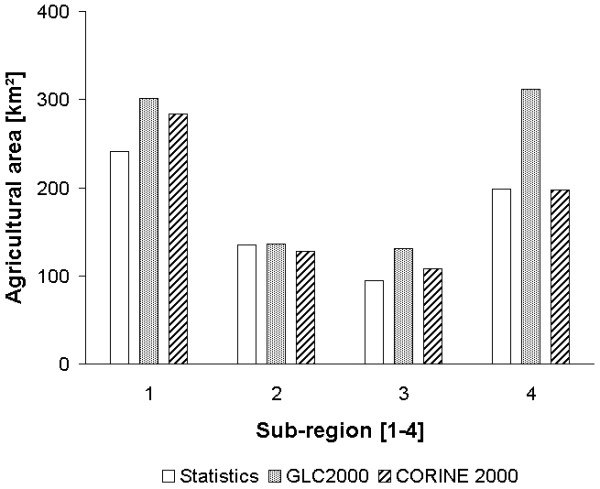
**Validation of Land cover land use products**. Comparison of agricultural areas described by statistical sources [30,42], GLC2000 and CORINE 2000 in km^2 ^for the four sub-regions of the Marchfeld region.

A reason for investigating the influence of different land cover classifications (GLC2000 versus CORINE 2000) is the higher spatial resolution of CORINE 2000. It is expected that CORINE 2000 represents the small scale land use structure of the Marchfeld region better than the GLC2000 classification. In Figure 3 the agricultural areas reported in the statistical source [30,42], the GLC2000 and CORINE 2000 are presented for all four Marchfeld sub-regions.

The agricultural areas presented in GLC2000 and CORINE 2000 have been computed using GIS tools. As shown in Figure 3, the GLC2000 considerably overestimates the agricultural areas (sub-regions one, three and four) by 25% to 57% compared to the statistical information. On the other hand, CORINE 2000 slightly over- (17%) or underestimates (6%) the agricultural areas compared to the statistical sources. However, approximately the same agricultural area is found for sub-region two for each land cover classification. For all sub-regions of the Marchfeld region the statistical data report an agricultural area of around 670 km^2 ^, GLC2000 of 881 km^2 ^, and CORINE 2000 of 718 km^2 ^. As the difference in agricultural area between CORINE 2000 and the statistical data is smaller than the difference between GLC2000 and the statistical data, we conclude that the CORINE 2000 land cover represents the real situation more precisely than GLC2000. The differences of the results described in Figure 2D and 2C showing an NPP decrease when changing from GLC2000 to CORINE 2000 can thus be explained by the fact that the BETHY/DLR model was driven for a smaller agricultural area.

**Figure 4:**
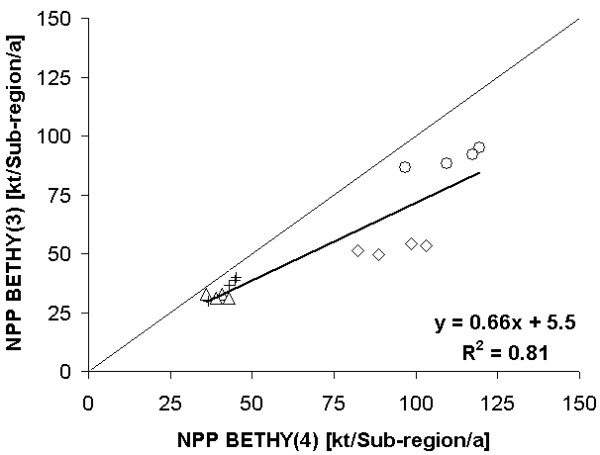
**Comparison of the BETHY/DLR model response to different land cover / land use products**. Comparison of the model results (NPP) of the BETHY/DLR runs BETHY(3) and BETHY(4) for the four Marchfeld sub-regions and the period 2000 to 2003. The nomenclature follows the scheme of table 3. Circles represent sub-region 1, triangles sub-region 2, crosses sub-region 3 and diamonds sub-region 4.

To proof this, the results for BETHY(3) and BETHY(4) are presented in Figure 4 as a linear correlation. For both model setups meteorology was fix (ECMWF), but the land cover classification was changed. With this direct comparison it becomes clear that the reason for the highly different model results presented in Figure 2C and 2D lays in the uncertainty in the two land covers.

**Figure 5:**
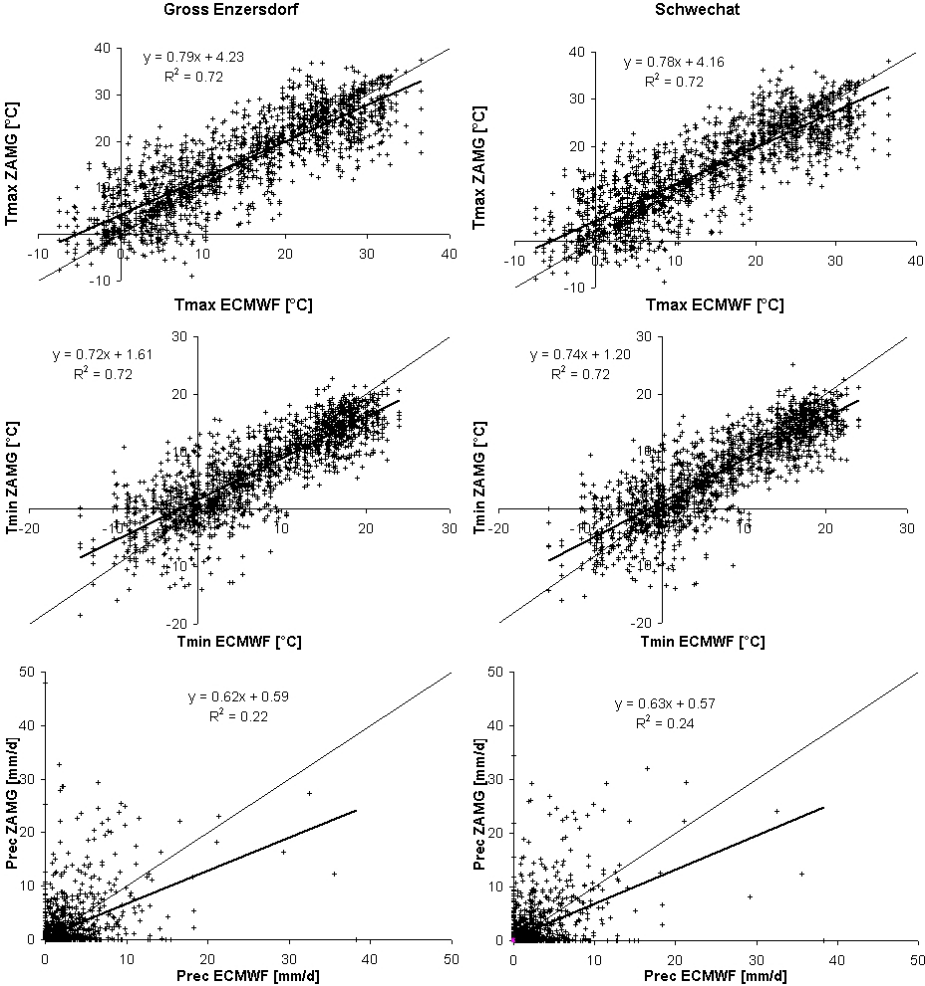
**Validation of ECMWF meteorology**. Comparison of the ECMWF time series of minimum and maximum temperatures as well as precipitation with the corresponding daily measured data of the ZAMG stations Gross Enzersdorf and Schwechat in the period 2000 to 2003.

When comparing the ECMWF data with the measured ZAMG data it is obvious that the ECMWF data underestimates the maximum and minimum temperatures (see Figure 5). The comparison of daily weather measurements is conducted for two of the 34 ZAMG weather stations which are situated closest to the Marchfeld (Schwechat and Gross Enzersdorf) and for the time period 2000 to 2003.

For both stations the maximum temperature of the ECMWF data is underestimated by about 21% expressed by a high coefficient of correlation of up to 0.72. The minimum temperature is underestimated even slightly higher (up to 28%) but again combined with a high coefficient of correlation (up to 0.74). In contrast, precipitation is not represented very well by ECMWF data as the correlation reveals high uncertainties. Hence a comparison of the ECMWF data for only two measurement stations is not very meaningful. Therefore the analysis has been expanded to all of the 34 available ZAMG weather stations. The analysis shows that the mean maximum and minimum temperatures of ECMWF data averaged over daily values in the period 2000 to 2003 are about 24% and 29% lower, respectively, than the temperatures recorded by the 34 ZAMG weather stations. However, minimum and maximum temperatures are both linked with a coefficient of determination of about 0.65, which is in good correspondence with the two presented observation stations in Figure 5. The comparison between sums of annual precipitation between the ECMWF and the ZAMG data reveals over- and underestimations of up to 90% for single stations. The daily precipitation rates averaged over all ZAMG observation stations show a coefficient of determination of about 0.27. This very low coefficient corresponds with the presented stations in Figure 5 and indicates poor agreement of measured and simulated precipitation.

As ECMWF data significantly underestimate temperature, the increase of NPP when using ZAMG data could be explained by longer vegetation periods in the ZAMG data. We investigated the vegetation period by computing the growing-degree-days (GDD). The basic equation is: GDD = [(T_MAX _+ T_MIN_)/2]-T_Base_, where T_MAX _and T_MIN _are daily maximum and minimum temperatures, respectively and T_BASE _is the base temperature which can be fixed at 10°C [43]. Furthermore, the growing period in Austria is assumed to be from mid March to mid October. The mean GDD averaged over all 34 ZAMG stations in Austria and the years 2000 to 2003 is about 1186.2, which is about 136.1 (~11.5%) more than the corresponding ECMWF GDD value (1050.1).

In a third model setup both models are driven with the reallocated ZAMG data to test the model response to homogenized trend data. Figure 2B and 2E show that both, the EPIC and the BETHY/DLR models respond in a consistent way, concerning their annual variability, to the reallocated ZAMG data. The variability in the NPP over the four years within one sub-region is about 4% (EPIC) and 3% (BETHY/DLR), respectively.

**Figure 6:**
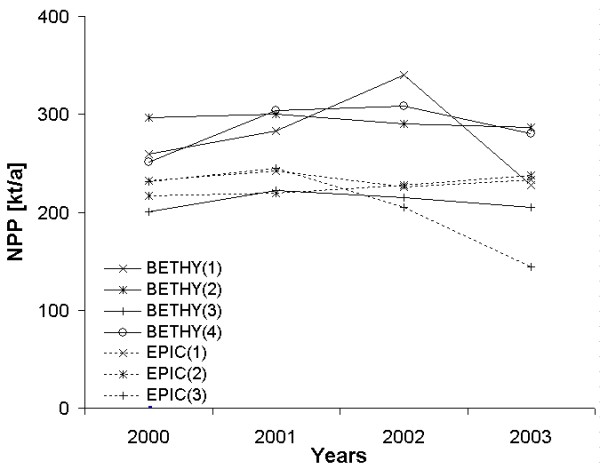
**Annual sums of NPP for the study area**. Annual sums of NPP in kilotonnes carbon for the Marchfeld region in the period 2000 to 2003 simulated with the models BETHY/DLR and EPIC. The nomenclature follows the scheme of table 2.

To give information about the annual variability of NPP within the model results, annual sums of NPP over the whole area of investigation are presented in Figure 6. The values are given in kilotonnes carbon per year.

The nomenclature in Figure 6 follows the scheme of table 2. When using the reallocated weather data, the annual variability of NPP is very low for both models BETHY(2) and EPIC(2), which can also be seen from Figure 2B and 2E. This is not surprising since they represent trend data with lower inter-annual variability. When looking at the model setup for BETHY(1) with measured ZAMG data, BETHY/DLR strongly responds to the climate data. This is very prominent for the year 2003, for which a water stress situation for the Marchfeld region is reported [44]. In comparison to the NPP sum calculated for 2002, the annual NPP in 2003 is lower by about 23%. However, this model response cannot be seen in the EPIC output, which might be due to the reason that for one of the four climate clusters, which is representative for most of the area of the Marchfeld region, higher crop yields have been simulated especially for winter crops in 2003. With the use of ECMWF data in model setup EPIC(3), the EPIC output shows a massive NPP decrease in 2003 compared to 2002. This could again be explained with the lower GDD of the ECMWF. In addition, the ECMWF data represent around 8.5% less precipitation over the days which have been counted as GDD.

The reason for the non equidistant annual differences between the BETHY/DLR model runs BETHY(3) and BETHY(4) might be that the misclassified pixels of GLC2000 represent non agricultural areas which react in different ways to climate conditions than agricultural areas.

It is notable that the variability of the model outputs can be as large as 36% for BETHY/DLR and 39% for EPIC when changing major input datasets. Furthermore, it is remarkable that both models response similarly when using the same datasets. For instance, all three model setups with the ECMWF data show for all four sub-regions a relative increase of NPP from 2000 to 2001 followed by a decrease in 2002 and again in 2003.

## Conclusions

Net-Primary-Productivity (NPP) has been modelled using the SVAT model BETHY/DLR and the biophysical process model EPIC for the Austrian Marchfeld region and the period 2000 to 2003. Both models seem to be robust but respond differently on alternative input datasets (i.e. meteorological and land cover / land use data). We have used meteorological data from the ECMWF and the ZAMG as well as a reallocated dataset based on ZAMG weather observations. Land cover / land use information have been taken from the GLC2000 and the CORINE 2000 products. With these datasets, we have performed a variability analysis with the two models BETHY/DLR and EPIC with respect to their output responses. We show that lower NPP values were modelled when using ECMWF data as an input compared to ZAMG data. This is confirmed by both models. The reason is traced to the underestimation of the GDD of about 11.5% in the ECMWF data. We observe that both models respond similarly to changes in input data, albeit with a different magnitude. For single years, variabilities in the NPP of up to 36% for BETHY/DLR and of up to 39% for EPIC can occur with alternative input data.

Besides the variability analysis of alternative model input data sources, we have also analysed the accuracy of the input data. We have found that the GLC2000 land cover classification overestimates the agricultural area of the Marchfeld region by 24%, whereas the CORINE 2000 dataset overestimates land cover classification by only 7%. With this finding preference for land cover datasets with higher resolution is recommended. The ECMWF data has been compared with measured data from ZAMG. We have found high uncertainties in the daily precipitation and small ones in daily maximum and minimum temperatures, which is confirmed by other studies.

For further investigations in other regions, the finding of the bias in the ECMWF data should be taken into account and crosschecked with local weather station data. In addition, more detailed land cover products should be considered with respect to spatial resolution and reported land use practices. Thus whenever the models (or any model) are applied under new conditions, local data (if applicable) should be used for both input and result comparison.

This study shows that especially for process-based modelling approaches, not only comprehensive validation and calibration approaches need to be applied, but also knowledge of input data uncertainty and variability of the modelling results need to be assessed. Process-based models have a potentially valuable role for various applications. However their validity must be determined where possible, especially when used for decision making processes.

## List of abbreviations

BETHY/DLR: Biosphere Energy Transfer Hydrology Model; CORINE: Coordinated Information on the European Environment; CYCLOPES: Carbon Cycle and Change in Land Observational Products from an Ensemble of Satellites; ECHAM: European Centre Hamburg; ECMWF: European Centre for Medium-Range Weather Forecasts; EPIC: Environmental Policy Integrated Climate; ETOP: Earth Topography and Ocean Bathymetry Database; GDD: Growing Degree Day; GPP: Gross Primary Productivity; JSBACH: Jena Scheme of Atmosphere Biosphere Coupling in Hamburg; LAI: Leaf Area Index; LUE: Light Use Efficiency NPP: Net Primary Productivity; PAR: Photosynthetically Active Radiation; POSTEL: Pole d'Observation des Surfaces Continentales par Teledetection; WGS84: World Geodetic System 1984; SVAT: Soil-Vegetation-Atmosphere-Transfer; ZAMG: Central Institute for Meteorology and Geodynamics.

## Competing interests

The authors declare that they have no competing interests.

## Authors' contributions

MT had the idea, provided the first draft for the design of the study and was responsible for the BETHY/DLR's part. FS provided the idea of including the EPIC model outputs for the study and carefully reviewed the manuscript during each step of the production. IM, KG and ES contributed to the analysis with their expertise, provided literature and developing ideas. All authors read and approved the final version of the manuscript.
